# Serum progesterone measurement on the day of fresh embryo transfer and its correlation with pregnancy success rates: A prospective analysis

**DOI:** 10.1016/j.clinsp.2024.100511

**Published:** 2024-10-09

**Authors:** Carla Maria Franco Dias, Suelen Maria Parizotto Furlan, Rui Alberto Ferriani, Paula Andrea de Albuquerque Salles Navarro

**Affiliations:** Department of Gynecology and Obstetrics, Faculdade de Medicina de Ribeirão Preto, Universidade de São Paulo, Ribeirão Preto, SP, Brazil

**Keywords:** Assisted reproduction techniques, Controlled ovarian stimulation, Embryo transfer, Progesterone, Clinical pregnancy

## Abstract

Studies regarding serum Progesterone (P4) concentration and Clinical Pregnancy Rates (CPR) in fresh Embryo Transfer (ET) after Controlled Ovarian Stimulation Cycles (COS) remain inconclusive. To find a P4 cutoff point on fresh ET day associated with higher CPR, and to identify predictive factors of CPR and P4, the authors conducted a prospective cohort of 106 patients who underwent COS at a public IVF center. The luteal phase was supported with vaginal micronized progesterone (200 mg, 8/8h), beginning on oocyte retrieval day. The primary outcome was CPR beyond the 8^th^ week of pregnancy. A ROC curve was constructed to identify the best cutoff point correlated with higher CPR. Multivariate analysis evaluated predictive variables of CPR and P4 concentration. P4 levels showed no significant differences between pregnant and non-pregnant patients (67.12 ± 31.1 ng/mL vs. 64.17 ± 61.76, *p* = 0.7465). The cutoff point correlated with higher CPR was P4 ≥ 28.9 ng/mL (AUC 0.5654). Women's age (OR = 0.878; 95 % CI 0.774–0.995) and top-quality embryo transfer (OR = 2.89; 95 % CI 1.148–7.316) were associated with CPR. Women's age ≥ 40 years (OR = 0.0956; 95 % CI 0.0156–0.5851), poor response to COS (OR = 0.0964; 95 % CI 0.0155–0.5966), and follicles ≥ 10 mm (OR = 1.465; 95 % CI 1.013–2.117) were associated with the cutoff point. As the ROC curve was unsatisfactory, P4 ≥ 28.9 ng/mL should not be used to infer gestational success. In fresh ET, P4 concentration may merely reflect a woman's age and individual response to COS rather than being a reliable CPR predictor.

## Highlights

• Studies are inconclusive about the existence of a serum progesterone concentration correlated with higher clinical pregnancy rates in fresh embryo transfer after controlled ovarian stimulation cycles.
• In fresh embryo transfer cycles, serum progesterone concentration may merely reflect factors associated with a favorable gestational prognosis rather than being a direct predictor of clinical pregnancy.
• Caution is warranted when interpreting serum progesterone concentrations in fresh embryo transfer cycles, as there is no established cutoff point to justify additional exogenous progesterone supplementation for endometrial rescue or a freeze-all approach.

## Introduction

### Background

Progesterone is the main hormone of the luteal phase. Produced by the corpus luteum after ovulation, it promotes transient changes in the endometrium, making it receptive to embryo implantation.^1^ This hormone increases uterine vascularization, stimulates the secretory function of the endometrial glands, stabilizes the endometrium, and induces the production of nitric oxide by the decidua basalis, relaxing the muscles of the uterus.^2^, ^3^, ^4^, ^5^ Given its significant importance, progesterone is essential in the context of assisted reproduction.^6^

With the advent of assisted reproduction, many infertile and subfertile individuals have been able to realize their dream of procreation. Among the techniques used, Controlled Ovarian Stimulation (COS) is particularly noteworthy. The use of gonadotropins promotes multi follicular growth, increasing oocyte yield and, consequently, the number of embryos formed, thus enhancing the chances of Clinical Pregnancy (CP) and live births per initiated cycle.^1^ It is estimated that the pregnancy rate resulting from assisted reproduction treatment in patients under 34 years old, and with 1 to 2 years of infertility, is 36.7 %;^7^ this rate falls to 8.3 % when treatment is performed in natural cycles or with minimal ovarian stimulation.^8^

Although extremely advantageous, COS disrupts physiological hormonal mechanisms, leading to an endogenous progesterone profile that differs from that observed in the luteal phase of natural cycles.^9^ The behavior of progesterone after oocyte retrieval in COS cycles is not yet well understood, but it is suggested that deficient endogenous production occurs, leading to a shortened luteal phase.^10^ This defect during the luteal phase is attributed to the supra-physiological levels of steroids produced by the increased number of post-puncture corpora lutea, which directly inhibit the release of endogenous Luteinizing Hormone (LH) via negative feedback on the hypothalamic-pituitary axis.^11^, ^12^, ^13^

Indeed, LH levels are suppressed during the luteal phase in stimulated cycles, regardless of the pituitary blockade protocol adopted.^2^^,^^12^^,^^14^^,^^15^ Protocols involving long-acting Gonadotropin-Releasing Hormone (GnRH) agonists promote the depletion of LH stores, with pituitary production resuming only 2 to 3 weeks after their discontinuation.^16^ On the other hand, despite faster pituitary recovery, cycles with GnRH antagonists also demonstrate premature luteolysis, resulting in worse gestational outcomes in the absence of Luteal Phase Support (LPS).^13^^,^^15^

In view of the poor gestational outcomes associated with the mechanism of premature luteolysis, supplementation with exogenous progesterone as LPS is routinely used in cycles with fresh Embryo Transfer (ET).^17^ However, studies specifying the concentration of serum P4 concentrations in COS cycles with fresh ET are scarce and inconclusive regarding the correlation between such levels and the prediction of gestational success.^6^^,^^18^, ^19^, ^20^, ^21^, ^22^ Since the ideal P4 concentrations after COS are not well established, it is common practice in assisted reproduction services to prescribe a standard regimen of exogenous progesterone starting on the day of oocyte retrieval, without considering the specific medications used, the ovarian response to COS, or the actual concentrations of P4.

Some studies have shown that both low and high P4 concentrations on the day of fresh ET may be associated with lower CP rates and live births after COS for In Vitro Fertilization (IVF) or intracytoplasmic sperm injection (ICSI).^22^ Furthermore, little is known about which factors potentially influence P4 levels in cycles stimulated with gonadotropins. These factors need further elucidation to optimize the results of Assisted Reproduction Techniques (ART) and to allow for the appropriate individualization of LPS in stimulated cycles, if applicable.

### Objectives

In this prospective study, the authors evaluated the optimal cutoff point for P4 concentration, measured on the day of fresh ET, that correlates with higher Clinical Pregnancy Rates (CPR) after cycles of COS. Additionally, the authors assessed potential factors related to P4 concentration and the occurrence of CP in gonadotropin-stimulated cycles followed by fresh ET, with luteal phase support provided using micronized vaginal progesterone.

## Methods

### Compliance with ethical standards

The present research project was approved by the Research Ethics Committee of the Clinics Hospital of the Ribeirão Preto Medical School – CEP FMRP USP (October 20, 2021 – CAAE n° 52042621.9.0000.5440). The study was conducted in accordance with the principles of the Declaration of Helsinki. Informed consent was obtained from all individual participants included in the study.

### Study design and settings

This prospective cohort study was carried out at the Human Reproduction Center of the Clinics Hospital of the Ribeirão Preto Medical School, University of São Paulo (HC FMRP-USP), a public IVF center in southeastern Brazil, from August 2021 to October 2023. All eligible patients who underwent COS with gonadotropins for fresh embryo transfer were invited to participate in the study. The primary outcome was Clinical Pregnancy (CP) beyond the 8^th^ week of gestation. All collected data were obtained through the analysis of the service's medical records.

### Participants

Patients were recruited to participate in the study on the first day of COS for the IVF/ICSI cycle. Individuals under 45 years of age with a Body Mass Index (BMI) below 35 kg/m^2^, who were scheduled to undergo COS followed by fresh ET, were considered eligible for inclusion. The participants were monitored throughout COS, ovarian puncture, and the luteal phase, and had their serum P4 levels measured on the day of fresh ET. Those included in the study were followed up until the day they took the pregnancy test (beta-hCG), performed 14 days after ET. Patients with a positive pregnancy test were further evaluated until the first Transvaginal Ultrasound (TVUS) examination to confirm clinical pregnancy at 8 weeks of gestation.

The exclusion criteria were as follows: cycle cancellation due to lack of response to COS; cancellation of fresh ET due to ultrasonographic abnormalities identified during COS (e.g., endometrial polyp, hydrosalpinx, unsatisfactory endometrium); serum P4 levels on the day of triggering ≥ 1.5 ng/mL or evidence of ovulatory signs on the day of trigger indication; follicular maturation triggering with a GnRH agonist; absence of retrieved oocytes or embryo(s) formed; use of dihydrogesterone (Duphaston®) for luteal phase support; and withdrawal from participating in the study or discontinuation of treatment.

### Methodology and cycle variables

The first TVUS for COS was performed on the second or third day of the natural cycle, or 5 days after stopping the combined oral contraceptive used for cycle programming in patients on the long GnRH agonist protocol. The initial medication doses were determined based on age, patient classification (normorresponder, poor responder, or hyperresponder), the number of antral follicles on the first TVUS, and BMI. Different types of COS protocols were used: flexible GnRH antagonist (ant-GnRH), long GnRH agonist (a-GnRH), and minimal stimulation (MILD).

In the ant-GnRH protocol, gonadotropins (150–300 IU/day) were administered during the first five days of COS, with the daily dose adjusted according to follicular growth as of the sixth day of stimulation. Ant-GnRH (ganirelix or cetrorelix, 0.25 mg/day) was started when the average diameter of the largest follicle reached ≥ 14 mm and continued until the day before or the day of follicular maturation triggering.

Meanwhile, in the a-GnRH protocol, a-GnRH was initiated during the mid-luteal phase of the previous cycle, followed by gonadotropin use (150‒300 IU/day) during the first six days of COS. The daily dose of gonadotropins was then adjusted according to follicular growth and continued until the day before or the day of follicular maturation triggering.

The MILD protocol (clomiphene citrate plus gonadotropins and ant-GnRH) was offered to women at risk of poor response to COS (poor responders or patients with antral follicle counts < 5). Clomiphene citrate (100 mg/day) was administered during the first five days of COS, and menotropin (Menopur®, 150 IU/day) was given on days two and four, and daily from day six onwards. Ant-GnRH (ganirelix or cetrorelix, 0.25 mg/day) was started when the average diameter of the largest follicle reached ≥ 14 mm and continued until the day before or the day of follicular maturation triggering.

Ultrasound monitoring of follicular growth began on the seventh (a-GnRH protocol) or sixth day (ant-GnRH protocol) of COS. Follicular maturation was triggered when one or more follicles reached an average diameter of ≥ 17 mm in patients at risk of poor response, and when three or more follicles reached an average diameter of ≥ 17 mm in the other patients. This was achieved using urinary hCG (10,000 IU, Choriomon®) or recombinant hCG (0.25 mg, Ovidrel®). In patients at high risk of Ovarian Hyperstimulation Syndrome (OHSS), characterized by the presence of 20 or more follicles with an average diameter of ≥11 mm on the day of triggering, an a-GnRH (triptorelin 0.1 mg/mL, 2 SC ampoules) was used to trigger follicular maturation. These patients were then excluded from the study, as all embryos were cryopreserved following the a-GnRH trigger, with no fresh transfer performed.

Oocyte retrieval was conducted 34 to 36 hours after follicular triggering. Luteal phase supplementation was performed with vaginal micronized progesterone (200 mg, 8/8 h, Utrogestan®) or dihydrogesterone (30 mg/day, Duphaston®), starting on the day of oocyte retrieval. Patients who received LPS with dihydrogesterone were excluded from the study.

Patients used micronized progesterone between 6:00 and 8:00 a.m. on the morning of the ET day. Peripheral blood was drawn at the Human Reproduction Center of HC FMRP-USP for P4 measurement on the day of fresh ET, between 9:00 and 11:00 a.m. Serum progesterone concentrations were measured using an immunoassay that employs direct chemiluminescence technology (Atellica IM PRGE®) with the Atellica IM Analyzer system from Siemens Healthineers. Samples with concentrations higher than 60 ng/mL were reanalyzed after dilution. The embryos were transferred at the cleavage stage (D2/D3) and blastocyst-stage (D5/D6). All patients included in the study were followed up until the date of the blood pregnancy test (beta-hCG), conducted 14 days after the ET, and, if positive, until the date of the first TVUS, performed at the service, at the 8^th^ week of gestation, to confirm CP.

The following individual patient parameters were evaluated: age (years); BMI (kg/m^2^); cause and duration of infertility; presence of thrombophilia; presence of genetic alterations; TSH level prior to embryo transfer; comorbidities; history of recurrent miscarriage (≥ 2 pregnancy losses before 20 weeks of gestation); Recurrent Implantation failure (RIF) (≥ 3 embryo transfers with negative beta-hCG); and poor response in a previous cycle (< 4 mature oocytes collected).

During the COS cycles for IVF/ICS and fresh ET, the following parameters were analyzed: total dose of gonadotropins; the number of days of stimulation; stimulation protocol; endometrial thickness on the day of triggering; the number of retrieved, mature, and fertilized oocytes; the number and morphology of formed and transferred embryos; and the number of vitrified embryos. Chemical and Clinical Pregnancy Rates (CPR) were also estimated.

### Bias

All eligible patients were invited to participate in the study and were followed up until the day of the pregnancy test (beta-hCG), and, if positive, until the day of TVUS for the clinical pregnancy assessment.

All obtained data were analyzed and presented.

Progesterone measurements were carried out by technicians who had no access to clinical information, or the type of treatment performed.

The researchers had no influence on the patient's treatment decisions, and the attending physicians did not have access to the results of the P4 concentrations measured on the day of fresh ET during the course of treatment.

### Study size

A convenience sample was analyzed, consisting of all eligible patients who underwent COS and fresh ET at the Human Reproduction Center of the Clinics Hospital of the Ribeirão Preto Medical School – USP, from August 2021 to October 2023.

### Quantitative variables

The P4 concentration on the day of ET was treated as a continuous variable. A Receiver Operating Characteristic (ROC) curve was constructed to determine a cutoff point for P4 concentration associated with higher CPR. This cutoff point was treated as a categorical variable.

The following parameters were treated both as continuous variables and as categorical variables: age (< 40 years old; ≥ 40 years old), BMI (non-obese: BMI < 30 kg/m^2^; obese: BMI ≥ 30 kg/m^2^), and the number of mature oocytes (MII) retrieved (< 4 MII: poor response to COS; ≥ 4 MII: absence of poor response).

### Statistical analyses

Statistical analyses were conducted using the SAS 9.4 program, with the significance level set at *p* < 0.05. Quantitative variables were summarized using measures of central tendency and dispersion, while qualitative variables were presented as absolute and relative frequencies. In order to compare quantitative variables in relation to the CP outcome, the Mann-Whitney non-parametric test was applied. For qualitative variables, the exact chi-square test was used to assess their association with CP.

Based on the obtained P4 values, an ROC curve was constructed to determine the best cutoff point correlated with higher CPR. Patients were categorized according to their P4 dosage and were divided into groups with P4 levels above and below the cutoff point. To compare quantitative variables relative to the P4 cutoff point, a Student's parametric *t*-test was applied. The exact chi-square test was also used to verify which qualitative variables were associated with the P4 cutoff point. Finally, a multivariate logistic regression model was constructed to determine which exploratory variables were predictive factors for the occurrence of CP and the P4 cutoff point.

## Results

### Participants

**Figure 1:**
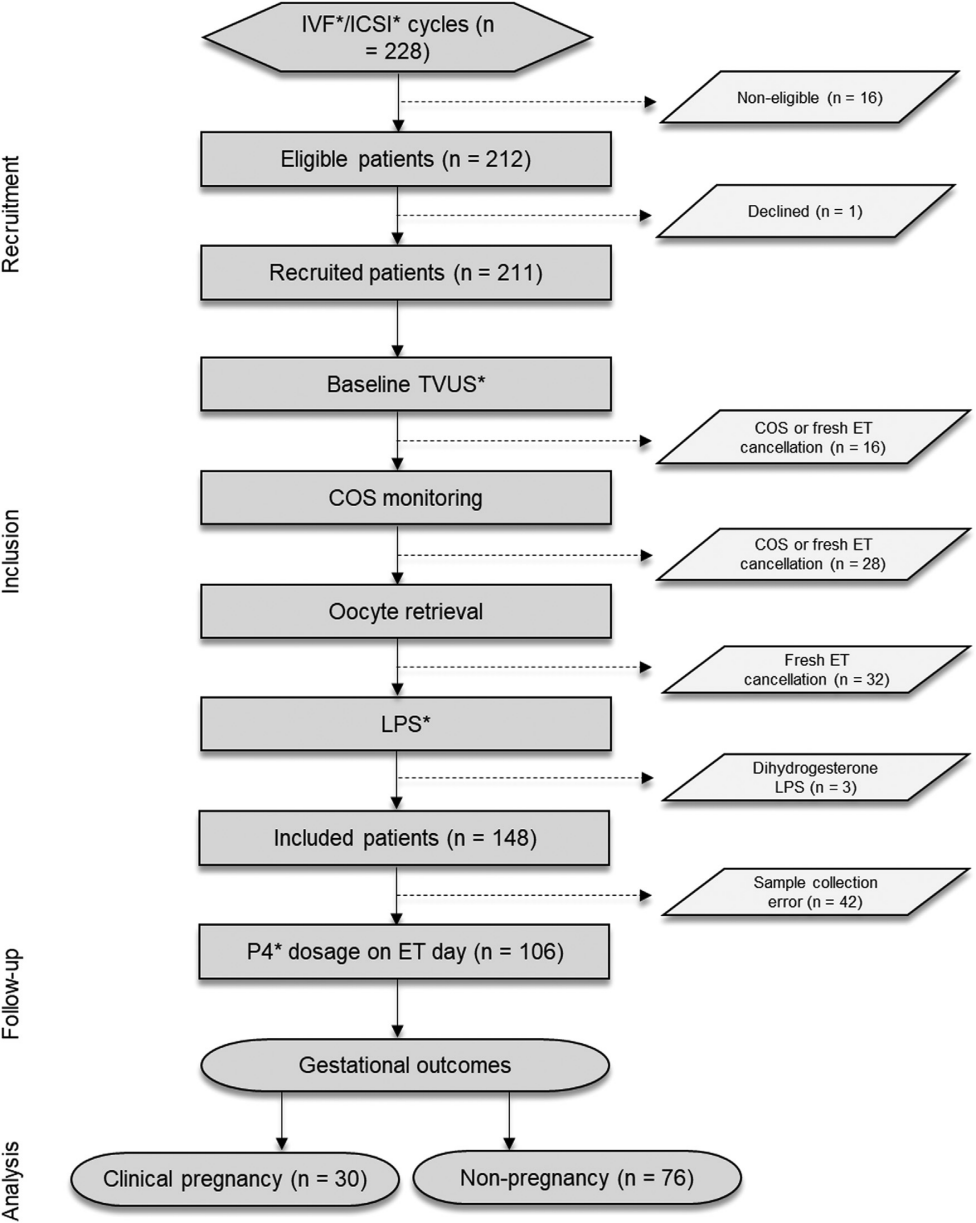
**Study flowchart.** COS, Controlled Ovarian Stimulation; ET, Embryo Transfer; ICSI, Intracytoplasmic Sperm Injection; IVF, In Vitro Fertilization; LPS, Luteal Phase Support; P4, Serum Progesterone; TVUS, Transvaginal Ultrasonography.

Two hundred and twenty-eight patients were scheduled to undergo IVF/ICSI cycles at the Human Reproduction Center of the Clinics Hospital of the Ribeirão Preto Medical School – USP, from August 2021 to October 2023. On the first day of COS, sixteen patients were deemed ineligible due to the cancellation of fresh ET for the following reasons: ovulation blockage with dihydrogesterone (Duphaston®) (n = 9); cycle cancellation due to a dominant follicle visualized on TVUS (n = 3); and a decision to freeze all embryos for Preimplantation Genetic Testing for Aneuploidies (PGT-A) (n = 4). Thus, 212 patients were considered eligible, and 211 consented to participate (Fig. 1).

During COS monitoring, 28 patients were excluded from the study based on the following criteria: abnormalities identified during TVUS monitoring, such as hydrosalpinx and endometrial polyps (n = 9); cycle cancellation due to poor response to COS (n = 2); cycle cancellation for personal reasons (n = 4); ovulatory signs on TVUS or P4 ≥ 1.5 ng/mL on the triggering day (n = 5); and triggering performed with an a-GnRH (n = 8). After oocyte retrieval, 32 patients were excluded due to the following reasons: absence of mature oocytes or embryos (n = 27) and cancellation of fresh ET for other individual reasons after ovarian puncture (n = 5). Following the initiation of LPS and scheduling of fresh ET, 3 patients were excluded because luteal supplementation was performed with dihydrogesterone (n = 3).

On the day of fresh ET, 42 patients did not have their P4 measured due to errors in blood collection or failure to collect the sample (n = 38) and other reasons (n = 4). Ultimately, 106 patients had their P4 levels evaluated on the day of fresh ET and were followed up until the gestational outcome.

### Descriptive data

Among the patients, who had a mean age of 35.9 ± 3.8 years, 69.8 % (n = 74) had primary infertility, 51.9 % (n = 55) did not have comorbidities, and 77.4 % (n = 82) were non-obese. The average duration of infertility observed was 6.75 ± 4.1 years. In the studied population, 5.7 % (n = 6) of women had a history of recurrent pregnancy loss, 23.6 % (n = 25) had shown a poor response to COS in a previous cycle, and 18.9 % (n = 20) had been diagnosed with RIF. The prevalence of infertility factors was as follows: male factor 63.2 % (n = 67); tubal obstruction 28.3 % (n = 30); low ovarian reserve 18.9 % (n = 20); leiomyomatosis 18.9 % (n = 20); advanced female age (≥ 40 years old) 16 % (n = 17); endometriosis 12.3 % (n = 10); Polycystic Ovary Syndrome (PCOS) 6.6 % (n = 7); and adenomyosis 5.7 % (n = 6). Only 9.4 % (n = 10) of the patients were diagnosed with unexplained infertility.

The average endometrial thickness prior to ET was 9.8 ± 2.24 mm. The majority of embryos transferred were at the cleavage stage 89.6 % (n = 95), and only 10.4 % (n = 11) were at the blastocyst stage. At least one embryo with top morphology was transferred in 55.7 % (n = 59) of cases, and in 57.6 % (n = 61) of the ETs, two embryos were transferred. The chemical pregnancy rate was 34.9 % (n = 37), and the CPR was 28.3 % (n = 30). The average P4 concentration found was 65.0 ± 54.7 ng/mL, with a minimum of 12.8 ng/mL and a maximum of 536.1 ng/mL.

### Outcome and main results

**Figure 2:**
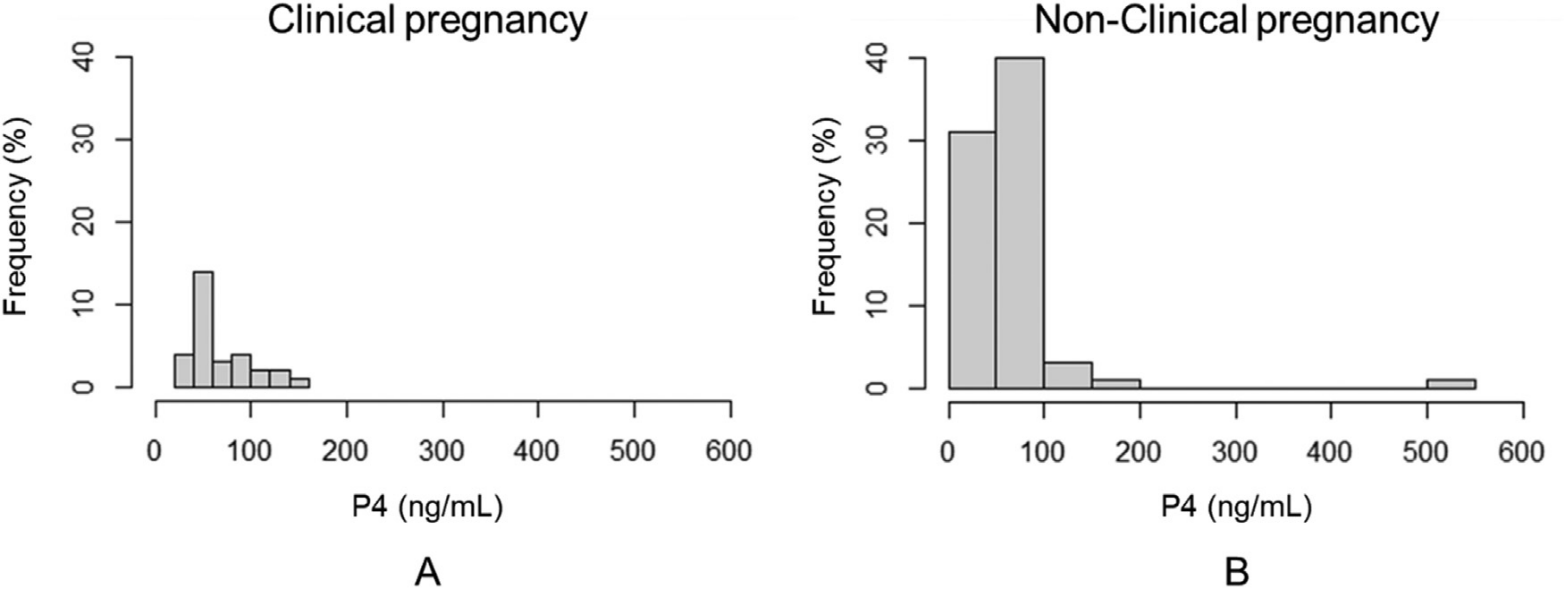
Comparison between P4 concentrations (ng/mL) among patients who became pregnant (A) and patients who did not (B).

The P4 concentrations measured on the day of fresh ET showed no significant differences between patients who achieved CP and those who did not (67.12 ± 31.1 ng/mL vs. 64.17 ± 61.76, p = 0.7465). Among the patients with CP, the minimum P4 level was 29.5 ng/mL, and the maximum was 146.47 ng/mL. As for the patients who did not become pregnant, the minimum P4 level was 12.77 ng/mL, and the maximum was 536.1 ng/mL (Fig. 2).

**Figure 3:**
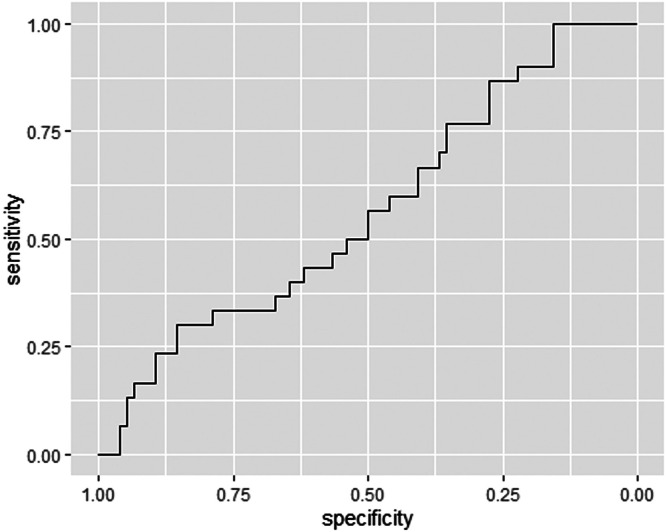
ROC curve for clinical pregnancy.

According to the constructed ROC curve, the best cutoff point for P4 concentration associated with higher CPR was 28.9 ng/mL. This cutoff point presented a sensitivity of 100 % and a specificity of 15.8 %, with an AUC of 0.5654 and a test power of 18.84 % (Fig. 3). The cutoff point showed a statistically significant correlation with the CPR (*p* = 0.0208). Since there were no patients who did not become pregnant with P4 ≥ 28.9 ng/mL, it was not possible to calculate the odds ratio.

Only the variables woman's age (OR = 0.878, 95 % CI 0.774–0.995; *p* = 0.0386) and top-quality embryo transfer (OR = 2.899, 95 % CI 1.148–7.316; *p* = 0.0214) showed a statistically significant correlation with CPR in the multivariate analysis. According to this result, it is observed that as female age increases, there is a reduction in CPR. Patients who had at least one Top-Quality Embryo (TQE) transferred had higher CPR than those without TQE transfer (37.3 % vs. 17 %, respectively). There were no statistically significant correlations between the other evaluated variables (Table 1).

**Table 1:** Comparison of variables based on the occurrence of clinical pregnancy.

Variables	Non-clinical pregnancy	Clinical pregnancy	*p-*value	Adjusted OR	95 % CI
N° of patients	76 (71.7 %)	30 (28.3 %)			
**Woman age (years)**	36.42±3.39	34.47±4.54	**0.0386**	**0.878**	**0.774‒0.995**
BMI (kg/m^2^)	26.78±4.41	26.62±3.80	0.8631		
Infertility duration (years)	6.91±4.20	6.33±4.01	0.5202		
Gonadotropin dose (IU)	2323.03±925.77	2140±660.84	0.2585		
COS duration (days)	10.16±2.12	10.50±2.45	0.4751		
Follicles ≥10 mm	8.32±3.95	9.63±5.58	0.2441		
N° retrieved oocytes	5.51±3.40	6.63±3.87	0.1453		
N° MII	4.16±2.56	5.23±2.88	0.0632		
N° fertilized oocytes	3.22±2.06	4.63±2.71	0.0046	1.366	0.913‒2.043
N° total embryos	2.67±1.81	3.73±2.41	0.0152	0.793	0.494‒1.273
N° top D2 embryos	0.88±1.30	1.80±1.79	0.0142	1.322	0.858‒2.036
N° top D3 embryos	0.79±1.32	1.13±1.36	0.2334		
N° extended-culture embryos	0.62±1.82	1.37±2.71	0.1712		
N° top D5 blastocysts	1.30±1.83	1.67±2.06	0.6862		
N° total blastocysts	1.75±1.91	2.78±2.05	0.2516		
N° vitrified embryos	0.59±0.95	1.63±1.79	0.0046	1.551	0.577‒4.170
Endometrium on trigger (mm)	9.72±2.31	10.03±2.08	0.5193		
Triple-layer endometrium	72 (94.7 %)	29 (96.7 %)	0.6729		
Previous pregnancy	23 (30.3 %)	9 (30 %)	0.9788		
Recurrent miscarriage	5 (6.6 % %)	1 (3.3 %)	0.5148		
Thrombophilia	2 (2.6 %)	0 (0 %)	0.3697		
Unexplained infertility	7 (9.2 %)	3 (10 %)	0.9003		
Male factor	49 (64.5 %)	18 (60 %)	0.6670		
Endometriosis	11 (14.5 %)	2 (6.7 %)	0.2697		
Tubal obstruction	20 (26.3 %)	10 (33.3 %)	0.4700		
Previous low ovarian reserve	16 (21.1 %)	4 (13.3)	0.3602		
Woman age ≥40 years	14 (18.4 %)	3 (10 %)	0.2872		
POS	4 (5.3 %)	3 (10 %)	0.3764		
Adenomyosis	6 (7.9 %)	0 (0 %)	0.1131		
Leiomyomatosis	14 (18.4 %)	6 (20 %)	0.8515		
Other infertility factor	5 (6.6 %)	4 (13.3 %)	0.2611		
Previous poor response	18 (26.7 %)	7 (23.3 %)	0.9694		
RIF	14 (18.4 %)	6 (20 %)	0.8515		
Low ovarian reserve COS	16 (21.1 %)	4 (13.3 %)	0.3602		
Comorbities	39 (51.3 %)	12 (40 %)	0.2936		
Poor response COS	27 (35.5 %)	9 (30 %)	0.5884		
Embryo stage transferred					
Cleavage-stage	70 (92.1 %)	25 (83.3 %)	0.1822		
Blastocyst-stage	6 (7.9 %)	5 (16.7 %)			
**Top-quality embryo transfer**	37 (48.7 %)	22 (73.3 %)	**0.0214**	**2.899**	**1.148‒7.316**
P4 ET (ng/mL)	64.17 ± 61.76	67.12±31.10	0.7465		

BMI, Body Mass Index; ET, Embryo Transfer; COS, Controlled Ovarian Stimulation; IU, International Units; kg, Kilograms; MII, Mature Oocytes; mL, Milliliters; mm, Millimeters; m^2^, square meters; ng, Nanograms; N°, Number; P4, Serum progesterone; POS, Polycystic Ovarian Syndrome; RIF, Recurrent Implantation Failure.

The number of follicles ≥ 10 mm on the day of trigger indication (OR = 1.465, 95 % CI 1.013–2.117; *p* = 0.0013) was considered a predictor for P4 levels above the cutoff point of 28.9 ng/mL. On the other hand, woman's age ≥ 40 years (OR = 0.0956, 95 % CI 0.0156–0.5851; *p* = 0.0007) and poor response to COS (OR = 0.0964, 95 % CI 0.0155–0.5966; *p* = 0.0014) were considered predictive variables for P4 levels below the cutoff point. It was not possible to calculate the odds ratio for the variable number of top blastocysts at D5 (*p* = 0.0029). Despite having a significant p-value (*p* < 0.05), the authors could not establish the type of association between this variable and P4 levels. The other variables did not show a statistical correlation with the cutoff point of P4 ≥ 28.9 ng/mL (Table 2).

**Table 2:** Comparison of variables based on serum P4 levels on the day of ET.

Variables	P4 < 28.9 ng/mL	P4 ≥ 28.9 ng/mL	*p*-value	Adjusted OR	95 % CI
N° patients	12 (11.3 %)	94 (88.7 %)			
Woman age (years)	37.50±3.83	35.7±3.8	0.1174		
BMI (kg/m^2^)	27.65±5.20	26.6±4.1	0.4272		
Infertility duration (years)	5.98±4.91	6.85±4.05	0.4997		
Gonadotropin dose (IU)	2087.50±997.47	2294.68±844.10	0.4346		
COS duration (days)	9.83±2.82	10.31±2.13	0.4854		
**Follicles ≥10 mm**	4.83±3.01	9.18±4.41	**0.0013**	**1.465**	**1.013‒2.117**
N° retrieved oocytes	3.25±2.45	6.16±3.55	0.0071	1.213	0.781‒1.885
N° MII	2.58±1.98	4.70±2.68	0.0095		
N° fertilized oocytes	2.00±1.13	3.83±2.37	0.0001		
N° cleaved embryos	2.00±1.13	3.35±2.20	0.0024	0.812	0.408‒1.616
N° total embryos	2.00±1.13	3.10±2.11	0.0103	1.522	0.938‒2.470
N° top D2 embryos	0.67±0.89	1.20±1.56	0.0906		
N° top D3 embryos	0.42±1.16	0.95±1.35	0.1960		
N° extended-culture embryos	0.42±1.00	0.88±2.22	0.2153		
**N° top D5 blastocysts**	0±0	1.65±1.93	**0.0029**	*	
N° total blastocysts	0.50±0.71	2.37±2.01	0.2149		
N° vitrified embryos	0.08±0.29	0.99±1.37	<0.0001	6.147	0.898‒42.086
Endometrium on trigger (mm)	9.53±2.37	9.84±2.24	0.6442		
Triple-layer endometrium	9 (75 %)	92 (97.9 %)	0.0004	68.965	0.5083‒90.909
Previous pregnancy	6 (50 %)	26 (27.7 %)	0.1124		
Recurrent miscarriage	2 (16.7 %)	4 (4.3 %)	0.0798		
Thrombophilia	0 (0 %)	2 (2.1 %)	0.6100		
Unexplained infertility	0 (0 %)	10 (10.6 %)	0.2351		
Male factor	6 (50 %)	61 (64.9 %)	0.3137		
Endometriosis	1 (8.3 %)	12 (12.8 %)	0.6593		
Tubal obstruction	6 (50 %)	24 (25.5 %)	0.0764		
Previous low ovarian reserve	5 (41.7 %)	15 (15.9 %)	0.0321	0.639	0.076‒53.475
Woman age ≥40 years	6 (50 %)	11 (11.7 %)	**0.0007**	**0.0956**	**0.0156‒0.5851**
POS	0 (0 %)	7 (7.5 %)	0.3280		
Adenomyosis	1 (8.3 %)	5 (5.3 %)	0.6705		
Leiomyomatosis	3 (25 %)	17 (18.1 %)	0.5643		
Other infertility factor	2 (16.7 %)	7 (7.5 %)	0.2806		
Previous poor response	3 (25 %)	22 (23.4 %)	0.9024		
RIF	1 (8.3 %)	19 (20.2 %)	0.3219		
Low ovarian reserve COS	5 (41.7 %)	15 (15.9 %)	0.0321	0.6988	0.0937‒52.083
Comorbities	6 (50 %)	45 (47.9 %)	0.8895		
Poor response COS	9 (75 %)	27 (28.7 %)	**0.0014**	**0.0964**	**0.0155‒0.5966**
Embryo stage transferred					
Cleavage-stage	12 (100 %)	83 (88.3 %)	0.2107		
Blastocyst-stage	0 (0 %)	11 (11.7 %)			
**Top-quality embryo transfer**	6 (50 %)	53 (56.4 %)	0.6751		
**Clinical pregnancy**	0 (0 %)	30 (31.9 %)	**0.0208**	*	

BMI, Body Mass Index; ET, Embryo Transfer; COS, Controlled Ovarian Stimulation; IU, International Units; kg, Kilograms; MII, Mature Oocytes; mL, Milliliters; mm, Millimeters; m^2^, Square meters; ng, Nanograms; N°, Number; P4, Serum progesterone; POS, Polycystic Ovarian Syndrome; RIF, Recurrent Implantation Failure.

When comparing the outcomes CP and P4 ≥ 28.9 ng/mL, the authors observed that both had a woman's age as a predictor of lower chances of pregnancy success and a lower P4 dosage on the day of fresh ET.

## Discussion

Studies regarding the existence of a P4 concentration correlated with higher CPR in fresh ET following COS cycles and LPS with exogenous progesterone remain inconclusive. It is known that there is significant variation in P4 levels between patients, even after performing COS with similar triggering and LPS in cycles with fresh ET.^23^ However, few studies describe the P4 profile in stimulated cycles and the factors affecting its concentration.^6^^,^^18^, ^19^, ^20^, ^21^, ^22^

In the present study, the constructed ROC curve detected higher CPR associated with the cutoff point of P4 ≥28.9 ng/mL, with a statistical correlation (*p* = 0.0208). However, despite the high sensitivity (100 %), this cutoff point proved to be non-specific (specificity = 15.8 %), and the obtained ROC curve had an AUC of 0.5654 and a test power of 18.84 %, which are considered unsatisfactory. Given the weak association found, it seems that correlating P4 concentration on the day of fresh ET with CPR may not be feasible.

Supporting these findings, previous studies have found comparable results,^19^^,^^24^, ^25^, ^26^, ^27^ demonstrating that P4 levels in fresh ET cycles are similar between pregnant and non-pregnant women. An example worth mentioning is the study by Rozen et al. (2022),^19^ a retrospective cohort of 825 unselected patients who underwent IVF/ICSI followed by a single fresh blastocyst ET. In that study, COS was carried out using an ant-GnRH protocol plus hCG triggering, and LPS was administered with vaginal progesterone (Utrogestan® 600 mg/daily or Crinone® 90 mg). After performing different types of analysis regarding P4 concentration on the day of ET, the authors found no statistical association between gestational outcomes – positive hCG, CPR, Live Birth Rate (LBR), or miscarriage. Furthermore, the AUC of the ROC curves for P4 in centile groups and as a continuous variable were 0.56 and 0.52, respectively, indicating that P4 levels were not predictive of gestational success.

Contrary to the present findings, some studies report an association between P4 levels and gestational success in stimulated cycles.^6^^,^^20^, ^21^, ^22^ Thomsen et al. (2018)^22^ were among the first authors to describe a non-linear relationship between P4 concentration on the day of fresh ET and gestational outcomes. In their prospective cohort study, 602 women who underwent P4 measurement on the day of fresh ET were categorized into P4 intervals as early luteal phase (ET at the cleavage stage) and mid-luteal phase (ET at the blastocyst-stage). In the early luteal phase, a P4 range of 60 to 100 nmoL/L (∼18.86 to 31.44 ng/mL) was correlated with higher chances of chemical pregnancy. As for the mid-luteal phase, a P4 range of 150 to 250 nmol/L (∼47.17 to 78.61 ng/mL) was associated with an optimal LBR. In that study, the authors claimed that P4 levels distinctly above these ranges (P4 > 400 nmoL/L [∼125.78 ng/mL]) were consistently linked to reduced gestational success.

Although these findings differ from ours, it is important to highlight some criticisms of the study by Thomsen et al. (2018).^22^^,^^28^ First of all, unlike this study, they did not construct an ROC curve to determine the ideal P4 values related to gestational outcomes, and the criteria used to establish the P4 ranges were not described. Secondly, their evaluation of P4 levels by percentiles (10^th^/50^th^/90^th^) and by quartiles (25^th^/50^th^/75^th^) showed no association with outcomes. Another notable flaw is that the adjusted estimated *odds ratio* for LBR did not reach statistical significance, raising doubts about the real clinical relevance of their findings. Finally, LPS varied among patients, complicating the replication of their study and introducing a risk of bias. All patients, regardless of whether they were triggered with hCG or a-GnRH, used vaginal micronized progesterone (300 mg/daily). Those triggered with a-GnRH also received a 1,500 IU bolus of hCG at Oocyte Pick-Up (OPU), and some patients received an additional bolus of hCG at OPU +5, based on their individual ovarian response to COS.

Another study with findings contrary to ours, conducted by Benmachiche et al. (2021),^20^ showed a statistically significant correlation between LBR and P4 levels measured in the mid-luteal phase (OPU +7) within the range of 41–60 ng/mL, after ET of cleavage-stage embryos (OR = 2.73; 95 % CI 1.29–5.78; *p* < 0.008). In their cohort study involving 328 women who underwent COS using an ant-GnRH protocol and a-GnRH triggering, all patients received a 1,500 IU bolus of hCG after OPU, along with vaginal micronized progesterone (600 mg/day) and oral estradiol (4 mg/day) as standard LPS. Despite these promising findings, the clinical applicability is limited, as most ETs are performed before OPU +7. At this point, it is too late to rescue endometrial receptiveness (if P4 levels are low), and the decision to *freeze all* embryos is no longer feasible (if P4 levels are high). Moreover, as embryo implantation occurs on D5/D6, the endogenous progesterone from embryonic hCG production introduces a risk of bias, leaving uncertainty regarding whether gestational success results from successful implantation or if implantation success is due to P4 levels being in optimal ranges.

Recent studies suggest that the debate regarding P4 concentration in the luteal phase of stimulated cycles is shifting towards defining what constitutes truly “low” or truly “high” P4 levels. Without a doubt, the importance of LPS with exogenous progesterone is clear.^29^, ^30^, ^31^ However, it remains debatable whether there is a definitive cutoff point below which additional progesterone supplementation is necessary to increase CPR and reduce the risk of miscarriage. Thomsen et al. (2018),^32^ described a P4 value < 60 nmoL/L (∼17.3 ng/mL) as a truly low value. Meanwhile, Petersen et al. (2018)^24^ found that the minimum P4 value on the day of fresh ET associated with the occurrence of live birth was 12.3 ng/mL. In the present study, the cutoff point was 28.9 ng/mL. However, as previously mentioned, studies on this topic are heterogeneous and limited in their ability to conclusively state that P4 concentration on the day of fresh ET is a reliable predictor of clinical pregnancy.

Regarding P4 concentration on the day of fresh ET, poor response to COS (OR = 0.0964, 95 % CI 0.0155–0.5966; *p* = 0.0014) and the number of follicles ≥ 10 mm on the triggering day (OR = 1.465, 95 % CI 1.013–2.117; *p* = 0.0013) were related to the progesterone cutoff found. Previous studies have reported similar associations between COS parameters and P4 concentration on the day of ET.^18^, ^19^, ^20^^,^^22^^,^^24^ For instance, Petersen et al. (2018)^24^ described a direct correlation between P4 dosage on the day of fresh blastocyst-stage ET and ovarian response, including the total number of follicles, number of oocytes retrieved, and estradiol concentration (*p* < 0.001). The authors analyzed a cohort of 600 patients who underwent ICSI with LPS performed using vaginal micronized progesterone (200 mg, 3×/day), similar to the present study.

Woman's age ≥ 40 years was a variable associated with P4 concentration below the cutoff point found (OR = 0.0956, 95 % CI 0.0156–0.5851; *p* = 0.0007). A comparable association between P4 levels on the day of fresh ET and age was described by Rozen et al. (2022),^19^ as previously mentioned. Other studies aimed at characterizing P4 concentration on the day of ET did not analyze this correlation.^6^^,^^18^^,^^20^, ^21^, ^22^^,^^24^ There is biological plausibility in these findings, given the abnormal luteal phase in stimulated cycles, where progesterone is produced at supraphysiological levels from multiple corpora lutea formed after OPU. As ovarian reserve tends to decrease with age,^33^ older women are expected to have poorer follicular growth and, therefore, lower serum P4 levels in the early and mid-luteal phases.^35^

The role of other variables that may influence P4 levels during the luteal phase of stimulated cycles and fresh ET must also be considered, such as individual factors related to endogenous progesterone production by the corpora lutea^23^ and the bioavailability of exogenous progesterone administered via different routes and metabolized individually.^36^, ^37^, ^38^, ^39^ Studies involving the transfer of thawed embryos with artificial preparation, and therefore without endogenous progesterone production, highlight other factors that may relate to interindividual differences in progesterone metabolism,^36^, ^37^, ^38^, ^39^ such as race, parity, and smoking.^40^ In the present study, several variables were evaluated, including BMI, the presence of comorbidities, total dose of gonadotropins, and the duration of COS, among others; however, none were found to have statistical relevance.

Moreover, in this study, the only variables considered as independent predictors of CP in the multivariate analysis were the woman's age (OR = 0.878, 95 % CI 0.774–0.995; *p* = 0.0386) and the transfer of at least one embryo with good morphology (OR = 2.899, 95 % CI 1.148–7.316; *p* = 0.0214). As demonstrated in several other studies,^20^^,^^41^, ^42^, ^43^ as female age increases, there is a reduction in CPR. The negative impact of aging on female fertility is well known, mainly due to the deterioration of oocyte quality and increased risk of aneuploidy.^34^ The European Society of Human Reproduction and Embryology (ESHRE)^41^ published a report in 2023, showing an overall CPR per ET after IVF and ICSI cycles of 34.6 % and 33.5 %, respectively, in 2019. At that time, women ≥ 40 years old had a CPR of 6.5 %. The Society of Assisted Reproductive Technology (SART CORS) reported that in 2021, in the United States of America, the LBR per oocyte retrieval in women under 35 years of age was 51.1 %, whereas in women above 42 years, the LBR was 3.9 %.^42^

As mentioned, the transfer of at least one top-quality embryo increased the chances of CP. Previous studies have also reported better gestational success rates associated with embryonic morphology.^44^, ^45^ Uyanik et al. (2023),^6^ in a prospective cohort study involving 340 patients who underwent fresh ET after COS, found a similar association between blastocyst morphology and pregnancy rates (OR = 5.686, 95 % CI 1.433–22.565; p = 0.013). Awadalla et al. (2021),^46^ in a retrospective study including 244 ETs (fresh and frozen), identified lower LBRs associated with poorer embryonic morphology and advanced female age. In younger women (25–32 years old), the LBR per embryo was 51 % for “good”, 39 % for “fair”, and 25 % for “poor quality” embryos. For older women (40–44 years old), the LBR per embryo was 22 % for “good”, 14 % for “regular”, and 8 % for “poor quality” embryos. Unfortunately, investigating the potential impact of P4 on fresh ET cycles with euploid embryos is challenging, as most services perform embryo biopsy followed by a *freeze-all* approach.

There are some important limitations to the present study. As an observational analysis, the assessed variables were extracted from patient medical records. Although it was a prospective cohort, and several efforts were made to minimize bias, as described in the Methods section, the evaluated sample size was small and there was a high rate of ET cancellations. Thus, studies with larger sample sizes are needed to confirm the present findings. The measurement of P4 was performed only once during the luteal phase – on the ET day. Despite potential variations throughout the day due to endogenous P4 pulsatility, a single measurement during the mid-luteal phase can provide insight into the degree of endometrial exposure to this hormone.^22^^,^^32^ Nevertheless, in an attempt to minimize the impact of circadian variation on endogenous progesterone, blood sample collection was standardized to a specific time (9:00 to 11:00 a.m.). Additionally, the day of ET varied (D2, D3, D5, and D6), and the results were not analyzed separately for embryos of different stages (blastocyst- and cleavage stage). However, it is noteworthy that in approximately 90 % of cases, the embryos were transferred at the cleavage stage, particularly on D3. Finally, with 16 % of the patients being aged ≥40 years, this may have affected the overall CPR, regardless of P4 concentrations.

## Conclusion

Although the authors identified a cutoff point for P4 concentration (P4 ≥28.9 ng/mL) associated with higher CPR, the ROC curve and the test power were unsatisfactory; therefore, this cutoff should not be used to predict gestational success. Additionally, the woman's age proved to be an independent predictive factor for both outcomes evaluated – CP and P4 measurement on the day of fresh ET. While embryo morphology was correlated with higher CPR, it was not associated with P4 concentration.

These findings suggest that P4 levels may merely reflect factors associated with a favorable gestational prognosis rather than being a direct predictor of CP. This hypothesis is supported by the observation that younger women are more likely to achieve clinical pregnancy, generally have better ovarian reserve, and respond better to COS, resulting in higher P4 levels after oocyte retrieval. Therefore, caution is warranted when interpreting P4 concentrations in fresh ET cycles.

In particular, cases with notably low P4 levels should be assessed individually, as there is no established threshold for defining such levels or consensus regarding the benefits of additional exogenous progesterone supplementation for endometrial rescue. There is also no defined cutoff point to justify canceling fresh ETs in favor of a *freeze-all* approach. Thus, if these findings are corroborated by studies with larger sample sizes, P4 measurement on the day of fresh ET may not be warranted.

## Funding information

Funding for this research was provided by the Foundation for Support to Teaching, Research, and Assistance of the Clinics Hospital of the Ribeirão Preto Medical School, University of São Paulo (FAEPA), and by the Department of Gynecology and Obstetrics of the Ribeirão Preto Medical School, University of São Paulo.

## Authors’ contributions

Carla Maria Franco Dias: Conceptualization; Investigation; Data curation; Formal analysis; Writing-original draft.

Suelen Maria Parizotto Furlan: Investigation; Writing-review & editing.

Rui Alberto Ferriani: Investigation; Writing-review & editing.

Paula Andrea de Albuquerque Salles Navarro: Conceptualization; Methodology; Supervision; Writing-review & editing.

## Conflicts of interest

All authors certify that they have no affiliations with or involvement in any organization or entity with financial or non-financial interests in the subject matter or materials discussed in this manuscript.

## References

[1] Lawrenz B, Coughlan C, Fatemi HM. (2019). Individualized luteal phase support. Curr Opin Obstet Gynecol.

[2] Fatemi HM, Popovic-Todorovic B, Papanikolaou E, Donoso P, Devroey P. (2007). An update of luteal phase support in stimulated IVF cycles. Hum Reprod Update.

[3] Bulletti C, de Ziegler D. (2005). Uterine contractility and embryo implantation. Curr OpinObstet Gynecol.

[4] Fanchin R, Righini C, Olivennes F, Taylor S, de Ziegler D, Frydman R. (1998). Uterine contractions at the time of embryo transfer alter pregnancy rates after in-vitro fertilization. Hum Reprod.

[5] Young SL, Savaris RF, Lessey BA, Sharkey AM, Balthazar U, Zaino RJ (2017). Effect of randomized serum progesterone concentration on secretory endometrial histologic development and gene expression. Hum Reprod.

[6] Uyanik E, Mumusoglu S, Polat M, Yarali Ozbek I, Esteves SC, Humaidan P (2023). A drop in serum progesterone from oocyte pick-up +3 days to +5 days in fresh blastocyst transfer, using hCG-trigger and standard luteal support, is associated with lower ongoing pregnancy rates. Hum Reprod.

[7] von Wolff M, Schwartz AK, Bitterlich N, Stute P, Fäh M. (2019). Only women's age and the duration of infertility are the prognostic factors for the success rate of natural cycle IVF. Arch Gynecol Obstet.

[8] Pelinck MJ, Vogel NE, Hoek A, Simons AH, Arts EG, Mochtar MH (2006). Cumulative pregnancy rates after three cycles of minimal stimulation IVF and results according to subfertility diagnosis: a multicentre cohort study. Hum Reprod.

[9] Fauser BC, de Jong D, Olivennes F, Wramsby H, Tay C, Itskovitz-Eldor J (2002). Endocrine profiles after triggering of final oocyte maturation with GnRH agonist after cotreatment with the GnRH antagonist ganirelix during ovarian hyperstimulation for in vitro fertilization. J Clin Endocrinol Metab.

[10] Zhao J, Hao J, Li Y. (2022). Individualized luteal phase support after fresh embryo transfer: unanswered questions, a review. Reprod Health.

[11] Bildik G, Akin N, Seyhan A, Esmaeilian Y, Yakin K, Keles I (2019). Luteal granulosa cells from natural cycles are more capable of maintaining their viability, steroidogenic activity and LH receptor expression than those of stimulated IVF cycles. Hum Reprod.

[12] Fatemi HM. (2009). The luteal phase after 3 decades of IVF: what do we know?. Reprod Biomed Online.

[13] Fauser BC, Devroey P. (2003). Reproductive biology and IVF: ovarian stimulation and luteal phase consequences. Trends Endocrinol Metab.

[14] Kolibianakis EM, Bourgain C, Platteau P, Albano C, Van Steirteghem AC, Devroey P. (2003). Abnormal endometrial development occurs during the luteal phase of nonsupplemented donor cycles treated with recombinant follicle-stimulating hormone and gonadotropin-releasing hormone antagonists. FertilSteril.

[15] Beckers NG, Macklon NS, Eijkemans MJ, Ludwig M, Felberbaum RE, Diedrich K (2003). Nonsupplemented luteal phase characteristics after the administration of recombinant human chorionic gonadotropin, recombinant luteinizing hormone, or gonadotropin-releasing hormone (GnRH) agonist to induce final oocyte maturation in in vitro fertilization patients after ovarian stimulation with recombinant follicle-stimulating hormone and GnRH antagonist cotreatment. J Clin Endocrinol Metab.

[16] Smitz J, Erard P, Camus M, Devroey P, Tournaye H, Wisanto A (1992). Pituitary gonadotrophin secretory capacity during the luteal phase in superovulation using GnRH-agonists and HMG in a desensitization or flare-up protocol. Hum Reprod.

[17] van der Linden M, Buckingham K, Farquhar C, Kremer JA, Metwally M. (2015). Luteal phase support for assisted reproduction cycles. Cochrane Database Syst Rev.

[18] Duport Percier M, Brouillet S, Mollevi C, Duraes M, Anahory T, Ranisavljevic N (2023). Serum progesterone concentration on pregnancy test day might predict ongoing pregnancy after controlled ovarian stimulation and fresh embryo transfer. Front Endocrinol (Lausanne).

[19] Rozen G, Rogers P, Mizrachi Y, Teh WT, Parmar C, Polyakov A. (2022). Serum progesterone concentration on the day of embryo transfer in stimulated cycles does not correlate with reproductive outcomes. Reprod Biomed Online.

[20] Benmachiche A, Benbouhedja S, Zoghmar A, Al Humaidan PSH. (2021). The impact of preovulatory versus midluteal serum progesterone level on live birth rates during fresh embryo transfer. PLoS One.

[21] Netter A, Mancini J, Buffat C, Agostini A, Perrin J, Courbiere B. (2019). Do early luteal serum progesterone levels predict the reproductive outcomes in IVF with oral dydrogesterone for luteal phase support?. PLoS One.

[22] Thomsen LH, Kesmodel US, Erb K, Bungum L, Pedersen D, Hauge B (2018). The impact of luteal serum progesterone levels on live birth rates-a prospective study of 602 IVF/ICSI cycles. Hum Reprod.

[23] Vuong LN, Ho TM, Pham TD, Ho VNA, Andersen CY, Humaidan P. (2020). The early luteal hormonal profile in IVF patients triggered with hCG. Hum Reprod.

[24] Petersen JF, Andersen AN, Klein BM, Helmgaard L, Arce JC. (2018). Luteal phase progesterone and oestradiol after ovarian stimulation: relation to response and prediction of pregnancy. Reprod Biomed Online.

[25] Howles CM, Macnamee MC, Edwards RG. (1987). Follicular development and early luteal function of conception and non-conceptional cycles after human in-vitro fertilization: endocrine correlates. Hum Reprod.

[26] Balasch J, Miró F, Burzaco I, Casamitjana R, Civico S, Ballescá JL (1995). The role of luteinizing hormone in human follicle development and oocyte fertility: evidence from in-vitro fertilization in a woman with long-standing hypogonadotrophic hypogonadism and using recombinant human follicle stimulating hormone. Hum Reprod.

[27] Hassiakos D, Toner JP, Muasher SJ, Jr Jones HW (1990). Implantation and pregnancy rates in relation to oestradiol and progesterone profiles in cycles with and without the use of gonadotrophin-releasing hormone agonist suppression. Hum Reprod.

[28] Venetis CA, Mol BW, Kolibianakis EM. (2018). Low as well as high serum P4 levels in the early and mid-luteal phase reduce the chance of a live birth following IVF treatment with fresh embryo transfer: where is the evidence?. Hum Reprod.

[29] Humaidan P, Bredkjaer HE, Bungum L, Bungum M, Grøndahl ML, Westergaard L (2005). GnRH agonist (buserelin) or hCG for ovulation induction in GnRH antagonist IVF/ICSI cycles: a prospective randomized study. Hum Reprod.

[30] Humaidan P, EjdrupBredkjaer H, Westergaard LG (2010). Yding Andersen C. 1,500 IU human chorionic gonadotropin administered at oocyte retrieval rescues the luteal phase when gonadotropin-releasing hormone agonist is used for ovulation induction: a prospective, randomized, controlled study. FertilSteril.

[31] Humaidan P, Thomsen LH (2013). Alsbjerg B. GnRHa trigger and modified luteal support with one bolus of hCG should be used with caution in extreme responder patients. Hum Reprod.

[32] Thomsen LH, Kesmodel US, Andersen CY, Humaidan P. (2018). Daytime Variation in Serum Progesterone During the Mid-Luteal Phase in Women Undergoing In Vitro Fertilization Treatment. Front Endocrinol (Lausanne).

[33] Taylor HS, Pal L, Seli E. (2019).

[34] Charalambous C, Webster A, Schuh M. (2023). Aneuploidy in mammalian oocytes and the impact of maternal ageing. Nat Rev Mol Cell Biol.

[35] Smith ADAC, Tilling K, Nelson SM, Lawlor DA. (2015). Live-Birth Rate Associated With Repeat In Vitro Fertilization Treatment Cycles. JAMA.

[36] Polat M, Mumusoglu S, Bozdag G, Ozbek IY, Humaidan P, Yarali H. (2020). Addition of intramuscular progesterone to vaginal progesterone in hormone replacement therapy in vitrified-warmed blastocyst transfer cycles. Reprod Biomed Online.

[37] Melo P, Chung Y, Pickering O, Price MJ, Fishel S, Khairy M (2021). Serum luteal phase progesterone in women undergoing frozen embryo transfer in assisted conception: a systematic review and meta-analysis. FertilSteril.

[38] Mumusoglu S, Polat M, Ozbek IY, Bozdag G, Papanikolaou EG, Esteves SC (2021). Preparation of the Endometrium for Frozen Embryo Transfer: A Systematic Review. Front Endocrinol (Lausanne).

[39] Ranisavljevic N, Huberlant S, Montagut M, Alonzo PM, Darné B, Languille S (2022). Low Luteal Serum Progesterone Levels Are Associated With Lower Ongoing Pregnancy and Live Birth Rates in ART: Systematic Review and Meta-Analyses. Front Endocrinol (Lausanne).

[40] Maignien C, Bourdon M, Marcellin L, Guibourdenche J, Chargui A, Patrat C (2022). Clinical factors associated with low serum progesterone levels on the day of frozen blastocyst transfer in hormonal replacement therapy cycles. Hum Reprod.

[41] European IVF Monitoring Consortium (EIM) for the European Society of Human Reproduction and Embryology (ESHRE); Smeenk J, Wyns C, De Geyter C, Kupka M, Bergh C, Cuevas Saiz I, De Neubourg D, Rezabek K, Tandler-Schneider A, Rugescu I, Goossens V. ART in Europe, 2019: results generated from European registries by ESHRE†. Hum Reprod. 2023 Dec 4;38(12):2321-2338. 10.1093/humrep/dead197. PMC10694409 37847771

[42] National Summary Report [Internet]. sartcorsonline.com. 2021. Available from: https://sartcorsonline.com/CSR/PublicSnapshotReport?ClinicPKID=0&reportingYear=2021.

[43] Wang SF, Seifer DB. (2024). Assessment of a Decade of Change in U.S. Assisted Reproductive Technology Cumulative Live-Birth Rates. Obstet Gynecol.

[44] Ahlström A, Westin C, Reismer E, Wikland M, Hardarson T. (2011). Trophectoderm morphology: an important parameter for predicting live birth after single blastocyst transfer. Hum Reprod.

[45] Vernon M, Stern JE, Ball GD, Wininger D, Mayer J, Racowsky C. (2011). Utility of the national embryo morphology data collection by the Society for Assisted Reproductive Technologies (SART): correlation between day-3 morphology grade and live-birth outcome. Fertil Steril.

[46] Awadalla M, Kim A, Vestal N, Ho J, Bendikson K. (2021). Effect of Age and Embryo Morphology on Live Birth Rate After Transfer of Unbiopsied Blastocysts. JBRA Assist Reprod.

